# Migrant Communities at the Center in Co-design of Health Literacy-Based Innovative Solutions for Non-communicable Diseases Prevention and Risk Reduction: Application of the OPtimising HEalth LIteracy and Access (Ophelia) Process

**DOI:** 10.3389/fpubh.2021.639405

**Published:** 2021-05-31

**Authors:** Sónia Dias, Ana Gama, Ana Catarina Maia, Maria J. Marques, Adalberto Campos Fernandes, Ana Rita Goes, Isabel Loureiro, Richard H. Osborne

**Affiliations:** ^1^NOVA National School of Public Health, Public Health Research Centre, Universidade NOVA de Lisboa, Comprehensive Health Research Centre, Lisboa, Portugal; ^2^Health Sciences Research Unit: Nursing (UICISA: E), Nursing School of Coimbra (ESEnfC), Coimbra, Portugal; ^3^Faculty of Health, Arts and Design, Centre of Global Health and Equity, Swinburne University of Technology, Melbourne, VIC, Australia; ^4^Department of Health Services Research, The University of Copenhagen, Copenhagen, Denmark

**Keywords:** migrant health, health literacy, co-design, non-communicable diseases, prevention, inequality, Ophelia, health literacy questionnaire

## Abstract

The drivers of high prevalence of non-communicable diseases (NCD) among migrants are well-documented. Health literacy is regarded as a potential tool to reduce health inequalities and improve migrant's access to and quality of health care. Yet, little is known about the health literacy needs among these groups and how to address them. This paper outlines the protocol for a migrant community-based co-design project that seeks to optimize health literacy, health promotion, and social cohesion in support of prevention of NCDs among migrants in Lisbon using the OPtismizing HEalth LIteracy and Access (Ophelia) process. This participatory implementation research project starts with a mixed-methods needs assessment covering health literacy strengths, weaknesses and needs of migrants, and local data about determinants of health behaviors, service engagement, and organizational responsiveness. Diverse migrant groups will be engaged and surveyed using the Health Literacy Questionnaire and questions on sociodemographic and economic characteristics, health status, use of health services, and perceived impact of the COVID-19 pandemic. Semi-structured interviews with migrants will also be conducted. Based on data collected, vignettes will be developed representing typical persons with diverse health literacy profiles. Migrants and stakeholders will participate in ideas generation workshops for depth co-creation discussions in simulated real-world situations based on the vignettes, to design health literacy-based multisectoral interventions. Selected interventions will be piloted through quality improvement cycles to ensure ongoing local refinements and ownership development. Through a genuine engagement, the project will evaluate the uptake, effectiveness and sustainability of the interventions. This protocol takes a grounded approach to produce evidence on real health literacy needs from the perspective of key stakeholders, especially migrants, and embodies strong potential for effective knowledge translation into innovative, locally relevant, culturally and context congruent solutions for prevention of NCDs among migrants. Given the diverse communities engaged, this protocol will likely be adaptable to other migrant groups in a wide range of contexts, particularly in European countries. The scale-up of interventions to similar contexts and populations will provide much needed evidence on how health literacy interventions can be developed and applied to reduce health inequality and improve health in diverse communities.

## Introduction

Non-communicable diseases (NCDs) are by far the leading cause of death globally (1). The European region is particularly affected by morbidity, mortality, and disability related to cardiovascular diseases, cancers, chronic respiratory diseases, and diabetes (2). A substantial amount of this burden is attributable to behavioral, dietary, environmental, and metabolic risk factors (3–5), which has called for attention to the need for improved prevention and treatment (6, 7).

NCD rates tend to be higher among communities that are disadvantaged and socially excluded, especially in high-income countries (6). Migrants are frequently among the most socially vulnerable populations, and they are disproportionally affected by NCDs compared to the host populations (8). In the recent years, migration to Europe has increased. In 2018, 22 million people residing in the European Union (EU) were born in non-EU countries, accounting for 4.4% of the total population (9). Although migrants in Europe appear to have lower prevalence of many NCDs on arrival compared with the host population—the so called “healthy-migrant effect”—morbidity rates, especially for obesity and cardiovascular disease, increase with longer duration of stay (8, 10). Throughout length of stay in the host countries, migrants in general tend to have higher incidence, prevalence and mortality rate for diabetes mellitus, ischaemic heart disease and stroke compared with the host population (10).

The NCD determinants behind health disparity among migrants are well-documented and include poorer socioeconomic conditions, reduced access to information and services, and adoption of unhealthy behaviors related to the new sociocultural contexts (8, 11). Migrants often face difficulties in accessing and using health services due to economic and legal constraints, lack of information about health rights and other individual, sociocultural, economic, administrative, and structural barriers (12, 13). As a result, they often tend to be missed by conventional disease prevention and surveillance programmes, to not receive health information and health education targeted to the mainstream population and to have delayed access to diagnosis and care (10, 14–16). These avoidable determinants impact on NCD care, generating excess downstream public health services costs, worsening NCDs outcomes, and increasing disadvantage and poverty (10).

Health literacy is of great importance to overcome these challenges by enabling people to build on over their own resources, such as previous knowledge and social connectedness. Health literacy is especially critical when working with disadvantaged groups who may lack access to and understanding of health information and health services (17). Health literacy is defined in a wide range of ways for different contexts (18, 19). It includes people's knowledge, confidence and comfort, which develop through daily activities, social interactions and across generations, to access, understand, appraise, remember and use information about health and health care for the health and well-being of themselves and those around them. Health literacy is regarded as a social determinant of health, and improved health literacy in itself is a goal of public health, being one of the key pillars of health promotion (20, 21).

Recent studies in Europe suggest that some members of migrant communities may have limited health literacy (22, 23). This means that these migrants can be unaware of a range of health information resources for prevention and other health promotion activities developed by local health professionals and health institutions (22–25). This situation may be aggravated by lack of awareness of their own rights and potential discrimination. Health literacy has been moving beyond an individualistic conception that focuses on a patient “deficit” or “risk approach,” toward a more social and contextualized perspective, as a dynamic social practice that develops in a context, is co-produced in social relations, depends on the resources at hand and, in many cases, is shaped by culture, personal experience, and knowledge (18, 26). This conception reinforces a strengths-based approach to health literacy, which is particularly relevant as many migrants have knowledge on health based on previous experiences and also passed on in their trusted social networks, but often are unaware of their health entitlements and how to navigate the health system (18).

In the NCD prevention context, a challenge has been to understand and meet the health literacy needs of the most disadvantaged and socially excluded populations. This is even more pertinent in the current context of the COVID-19 pandemic, where its adverse impacts are likely to aggravate social and health inequalities. The available evidence on the initial effects of the pandemic shows a disproportionate impact on migrants, especially in Southern European countries (27). In addition, access to health services has been constrained at expense of COVID-19 care. In these circumstances, the health literacy needs of populations, particularly those most underserved, become even more difficult to address.

In Portugal, as in Europe in general, little is known about health literacy and NCDs in most vulnerable populations, where many migrants are included. This knowledge is crucial to identify and understand the diversity of strengths, needs, and challenges in health literacy of population groups and allows the design and development of bespoke health literacy responses that optimize opportunities to improve equity in access to care (19).

The global and national commitments for improving health outcomes and well-being of all populations, ensuring that no communities are left behind, call for effective implementation of evidence-based interventions and comprehensive approaches. Within a co-design approach, the OPtismizing HEalth LIteracy and Access (Ophelia) process seeks to create, through genuine engagement and participation of community members and other relevant stakeholders, local fit-for-purpose health literacy solutions that address identified needs and taking into account their cultural specificities (19). The Ophelia process embodies a set of principles (see Supplementary Table 1) that guide the aims, development, and implementation of structured interventions to improve health and equity (28).

This paper outlines the protocol of a project for the co-creation of solutions that optimize health literacy, health promotion, and social cohesion in support of prevention of NCDs among migrant populations in Portugal using the Ophelia process. To our knowledge, this is the first project applying the Ophelia process with migrants in the EU. It will address the health entities limited knowledge on the bottom-up needs of migrants, especially given the large number of different cultural groups. By having the migrant communities at the front of the co-design process, in true partnership with other stakeholders, they will be central in developing, refining, and implementing meaningful solutions. Enabling the key recipients to take the control can be crucial to develop ownership, which may greatly assist in ensuring that feasible and scalable activities and interventions are developed.

## Methods and Analysis

The 3-year project “Health Literacy, Health Promotion and Social Cohesion for the Prevention of NCDs among Migrant Populations” was launched in January 2020 and gathers a multisectoral consortium of national and international academic institutions and renowned entities for social, health and community support.

**Figure 1:**
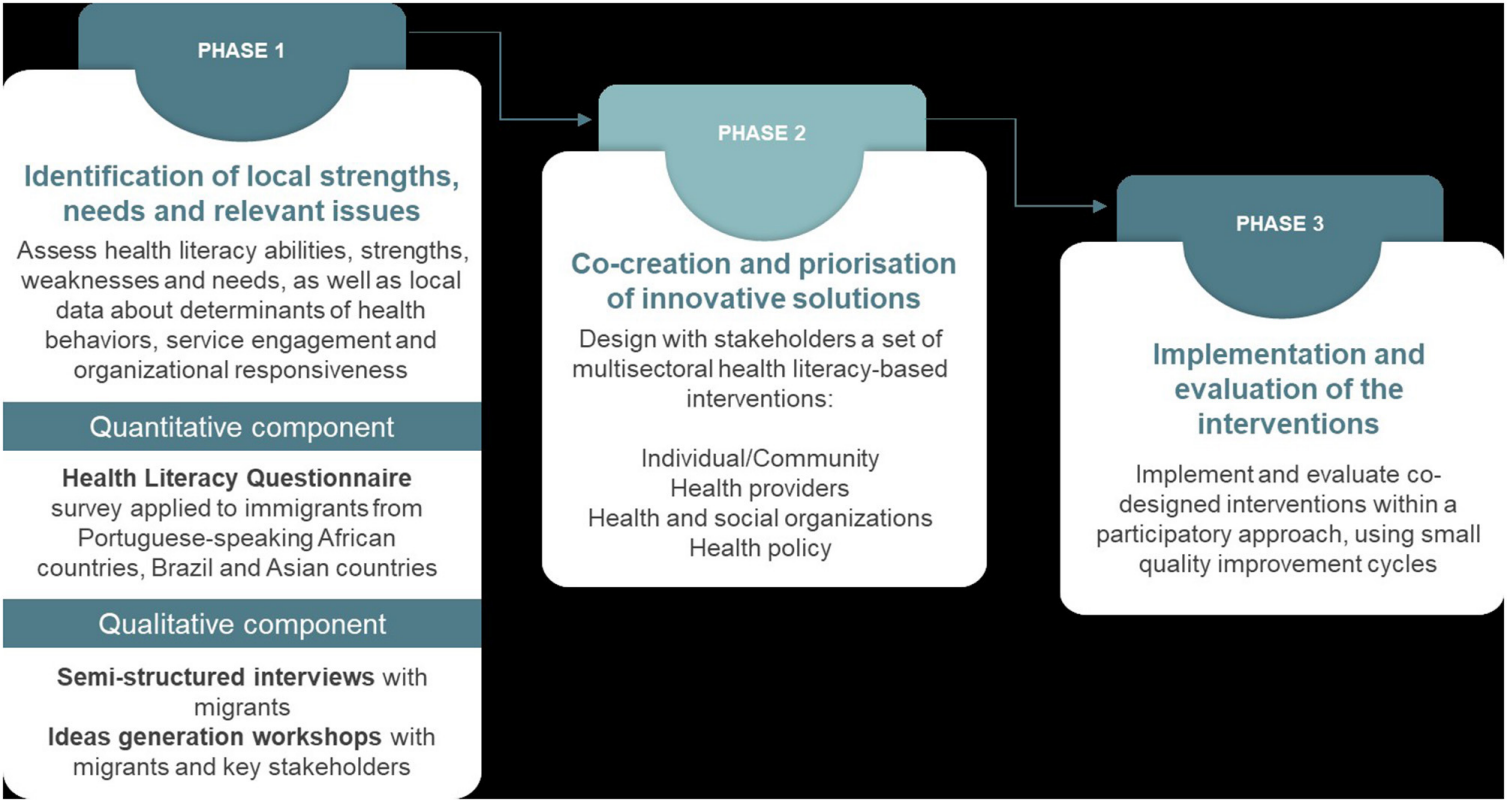
Phases of the project.

This project follows the Ophelia process (19). The first phase involves a needs assessment covering health literacy abilities, strengths, weaknesses and needs, as well as local data about determinants of health behaviors, service engagement, and organizational responsiveness (Figure 1). The second phase consists of co-designing with the migrant communities and other stakeholders a set of multisectoral interventions (covering individual/community, health providers, health and social organizations, and health policy). In the third phase, the co-designed interventions will be implemented and evaluated using quality improvement cycles followed by wider implementation (19).

A community-based participatory research approach is adopted, where relevant stakeholders (migrant communities, academia, health and social sectors, and policy-makers) are engaged actively throughout the different phases (29). Within this process, the project embodies implementation research—an innovative integrated approach linking research to action in order to produce useful evidence that is translatable into effective practices (30). Specifically, this process ensures the outputs are built *in situ* and are needed and wanted by not only the end user, but all the stakeholders involved in implementing and potentially scaling the derived interventions.

### Setting

The project is being implemented in the Lisbon district, where most of the foreign-born population in the country reside (31). In 2018, the foreign-born citizens represented 8.6% of the total population in Portugal (10,295,909 residents) and comprised 15.5% of the total population in the capital (Lisbon) (31–34). The most common nationalities are Brazilian, Cape Verdean, British (UK), Romanian, and Ukrainian, with Chinese, Indian, and Nepali nationalities being on the rise (31). Regular migrants (i.e., with authorized residence permit) in Portugal increased 22.9% from 2018 to 2019 (while no official data is available to account for undocumented migrants living in the country) (31).

Despite the acknowledgment that Portugal has some of the most inclusive health policies for migrants (35), research indicates an underuse of health services among some migrant groups, especially those most socially vulnerable, new arrivals, and undocumented migrants (14). Related barriers include lack of information on migrants' health rights and services available, language and cultural differences, stigma, economic constraints, and structural and administrative obstacles (36).

### Application of the Ophelia Process

#### Identifying Local Health Literacy Strengths, Needs, and Relevant Issues

The needs assessment comprises two components: a quantitative component to assess health literacy and identify factors hindering prevention, early diagnosis, and risk reduction of NCDs (including barriers to access and use of healthcare services); and a qualitative component intended to collect complementary information on locally relevant issues on health literacy, social support and integration, NCDs risk factors, prevention and care, services engagement, and organizational responsiveness.

##### Quantitative Component

The quantitative study consists of a survey conducted with a community-based sample of migrants. The IOM's definition of “migrant” is adopted in this protocol, referring to “a person who moves away from his or her place of usual residence, whether within a country or across an international border, temporarily or permanently, and for a variety of reasons” (37). The inclusion criteria include: being born in a Portuguese-speaking African country (PALOP), Brazil or an Asian country, being ≥18 years old, speaking Portuguese, English, Arabic, Bengali, Hindi, Mandarin or Nepali, residing in Portugal for 10 years or less, and currently residing in the Lisbon Metropolitan Area, regardless of migration status (i.e., regular, irregular, refugee, or asylum seeker). Individuals who visibly present unstable cognitive and/or emotional status and who are under effect of drugs and/or alcohol that may prevent them from being able to complete the survey are excluded from participation.

For the purpose of sample size calculation it was considered the total number of migrants residing in the Lisbon Metropolitan Area with legal resident status, which according to the data available was 240,963 in 2018 (38), and the existence of 50% of the characteristics under study (i.e., the worst case scenario, as the prevalence of the characteristics to be studied is not known), at a 95% confidence level and with a margin error rate of 5%. It was estimated that a minimum of 384 migrants will be surveyed, but efforts will be endeavored to enrol a higher number of participants of each origin group, in order to strengthen the robustness of statistical analysis and account for the diversity within groups. The percentage of the three origin groups under study was defined based on their distribution in the total migrant population. According to official data, migrants living in the Lisbon Metropolitan Area comprise 63,096 from PALOP, 50,312 from Brazil, and 39,887 from Asian countries (38). Thus, according to the respective proportion, at least 158 migrants from PALOP, 126 from Brazil, and 100 from Asian countries will be recruited.

Stakeholders, such as non-governmental and governmental organizations and migrant associations that work in proximity with migrant communities, were engaged in the project from the start. These partners have collaborating in publicizing the study within the communities and their networks and serving as recruitment sites, where attendees are approached and invited to participate in the study. Also, several informal leaders of the migrants' communities have been invited to collaborate as recruiters of potential participants within their social networks, and as interpreters.

Data will be collected using the Health Literacy Questionnaire (HLQ), available in Portuguese, English, Arabic, Bengali, Hindi, Mandarin, and Nepalese. The HLQ will be administered through an interview by trained bi-lingual researchers to ensure inclusion of people with low educational levels or that experience difficulties with self-administration.

The HLQ is a widely used measure that provides detailed insights into individual and community health literacy strengths and needs across nine distinct domains (see Supplementary Table 2), allowing the identification of “profiles” of communities (26, 39, 40). The HLQ has strong construct validity, reliability, and acceptability in several contexts and settings (41–46). In addition, data will be collected on demographics, socioeconomic, and health status (i.e., sex, age, marital status, country of origin, length of residence in Portugal, migration status, educational level, occupation, income level, fluency in Portuguese, self-perceived health status, chronic disease, and incapacity), and use of health services in Portugal (based on the National Health Survey), as well as on the perceived impact of the COVID-19 pandemic in health literacy, health behaviors and access to health services among participants.

Descriptive statistical analyses will be used to assess the health literacy levels of migrants within the nine domains of the HLQ. Computing the mean scores obtained in each dimension will allow to identify possible health literacy strengths and weaknesses in these populations. To understand the association between health literacy levels and the demographic, socioeconomic, and health condition factors that characterize migrants from the different regions of origin under study, a multiple linear regression analysis will be performed. Cluster analysis of HLQ data will be used to identify subgroups of migrants with similar health literacy profiles among the total sample and within each origin group.

##### Qualitative Component

The following step of the needs assessment consists in semi-structured individual interviews that will be conducted with migrants with NCD risk factors. The cluster analysis will be used to assist with maximum diversity sampling, as a guide to select migrants with diverse patterns of health literacy strengths and limitations, as well as from diverse demographic backgrounds. Up to four migrants per cluster will be interviewed. The main topics to explore in the interviews include the health background of the migrant in terms of risk factor profile (e.g., raised blood pressure and blood glucose, increased weight, family history of chronic disease, as well as behavioral risk factors), information and support they may have received from health entities. The perceived health literacy needs and the areas the participant feels confident about (identified respectively by the HLQ results on the domains with lower and higher scores) will also be explored.

Based on the data from the HLQ survey and the interviews, vignettes that represent typical persons across the clusters will be developed. As per the Ophelia manual (47), they will cover realistic descriptions of the health literacy strengths and weaknesses that influence individuals' ability to protect their health and interact with the health system.

Stakeholders will then be invited to ideas generation workshops where the vignettes are presented and discussed to generate ideas for ways to improve information and services for migrant groups at risk of NCDs. The workshops will last ~2.5 h and will be conducted *via* a web conferencing facility or face to face. There are four questions that guide the discussion: “Do you recognize people like this in your community?” or “Do you see people like this in your unit/service/organization?”; “What sorts of issues is this person facing?” or “What barriers to navigating the health system this persons may face?”; “What strategies could you use for an individual like this?”; and “What could your organization or community organization do if you had many attendees like this in your organization or community?”

Members of migrant communities, ranging from people recently arrived to successfully settled, as well as migrants with a wide range of health literacy strengths and challenges will be invited to take part in the ideas generation workshops. Relevant local stakeholders responsible for the provision of health care (health practitioners and managers from primary and secondary care), social care (including public, private, not-for-profit organizations), digital health experts, and a variety of other representatives from region-wide non-governmental organizations and local community-based organizations will also participate. Partners with expertise on network and digital health technologies and solutions will participate, given that the internet and social networks are increasingly two of the most important resources for the general population to search for health information and its integration can be of great added value. Overall, at least one workshop with each of the migrant origin groups and three workshops with stakeholders will take place. Each workshop will include ~5-10 participants. Participants will be recruited using a purposive sample of members of stakeholders' organizations and practitioners within their networks with experience on migrant health. The workshops will be conducted by the research team.

For the analysis of qualitative data, audio recordings of the interviews will be transcribed, and the transcripts will be analyzed through content analysis technique (48). This technique allows systematize the data in key topics and organize it in different categories and sub-categories (49).

#### Co-creation and Prioritization of Innovative Solutions

Representatives of key migrant groups and relevant local stakeholders responsible for the provision of health or social care to migrant communities in the Lisbon Metropolitan Area will be invited to participate in a set of workshops dedicated to prioritizing and refining the interventions. Overall, the prioritization outcomes will address different levels of the system, namely at individual, community, practitioner, and policy levels.

While it is not possible to predict the specific interventions, they are expected to include pathways and support for migrants' health promoting behaviors, including for adopting healthy lifestyles. They may also cover awareness of health services available, as well as increased information, skills, strategies, and tools to navigate in the local health services, engage and communicate with healthcare providers, and appraise practical support and health information. These potential outcomes will be refined throughout the partnership discussions, and even new outcomes may emerge during this step. Overall, the participatory process will help assure that the defined outcomes will be achieved and meet the real and unforeseen needs.

The research team will facilitate the multisectoral co-design process, including the design of the plans of implementation and evaluation of the selected interventions using a Programme Logic Model aligned with the Ophelia manual (47). The implementation plan will account for the expected challenges/risks related to the pandemic context and the respective measures to mitigate them. The evaluation plan will also consider the potential impacts of the COVID-19 pandemic in the expected outcomes.

A rapid review of scientific publications and gray literature will be also conducted to identify and map interventions that potentially tackle prevention of NCDs. The context of relevant studies will be noted and these data will also inform the prioritization of solutions to be implemented.

#### Implementation and Evaluation of the Interventions

In the third phase, a participatory implementation research of health literacy-informed solutions will be carried out. Throughout the piloting of the co-designed interventions, quality improvement cycles will be used, whereby organizations develop and implement trials, actively examine and evaluate the immediate and intermediate outcomes, and refine materials and processes to improve the effectiveness, local ownership, uptake and sustainability of the intervention. The health literacy outcomes associated with the tested interventions may be examined with some HLQ scales if relevant. Regular workshops will also take place to provide a venue for stakeholders implementing interventions to share ideas and resources, and to communicate key findings, as a community of practice.

## Discussion

This is a pioneering project that will address the health literacy needs of a population who tends to be disproportionately affected by health disparities. As documented in the literature, particularly socially vulnerable migrants tend to present poorer health outcomes than native populations, face increased difficulties in access to health services and, according to the limited evidence available, these populations tend to face increased health literacy challenges (10, 12, 22). Despite the global and national efforts to improve health of those most underserved, such as migrant groups, frequently these populations are understudied, and little involved in the efforts to identify their real needs. This project will assist with reducing this knowledge gap. It has been widely recognized that the promotion of health literacy can be a powerful tool to reduce health inequalities and improve access and quality of health care (26). The project can help to enhance migrant communities and stakeholders' capacity building for health literacy action. This is even more pressing in the current times of COVID-19 pandemic, where social inequalities in health and access to services have intensified, affecting asymmetrically those who are already disadvantaged (50).

This protocol follows the Ophelia process, a community co-design approach that is now being applied in many countries, that operationalizes the health literacy concept on a large scale and whose impacts have been well-documented (28, 40, 51–53). Indeed, Ophelia process has demonstrated to be a feasible approach by which organizations can develop tailored responses to the health literacy needs of their attendees/users, with positive outcomes such as changes in health literacy, behavior, knowledge, and management of risk factors of specific diseases (28, 51, 54–58). The project may provide useful insights related to health literacy research and practice in specific areas where migrant populations face disproportionate adverse impacts, which can inform global efforts on prevention of NCDs.

Through an implementation research that uses a participatory approach from the start, this project takes a systematic and grounded approach to produce evidence on real health literacy needs from the perspective of diverse key stakeholders, giving a particular attention to that of migrants. By developing a mixed-methods needs assessment—a quantitative HLQ survey combined with interviews with migrants and workshops with key stakeholders—we will be able to gather robust, rich and contextualized evidence on health literacy among these populations to inform researchers, health and social practitioners and decision makers. Indeed, this is even more pertinent in the context of the COVID-19 pandemic, where deepening social and health inequalities and increasing constraints in access to health services are resulting in new and unexpected needs. The fact that the needs assessment explores the impacts of the COVID-19 pandemic in health literacy, health behaviors and access to health services will assist in this effort.

Moreover, this project embodies a strong potential for effective knowledge translation into culturally and context congruent solutions that are locally relevant. The active involvement of several stakeholders in all phases of the project will allow to co-design and implement multisectoral interventions, reinforcing its potential for high impact in innovation generation and system reform. This participatory implementation research will pave the way to build and reinforce synergies between stakeholders, with exchange of knowledge and resources, enhancing empowerment of migrant communities, and increasing responsiveness of health and social organizations (59). Additionally, without working beyond the health sector it is not possible to effectively address the complex challenges that societies face in its efforts to improve health and well-being, and reduce inequalities (59). This approach not only helps to address health and well-being challenges that transcend traditional sectoral boundaries, but also promotes good governance by building accountability across sectors and encouraging broader participation in the policy process. Overall, this will potentially strengthen the sustainability of the project beyond its timeframe and contribute to reducing the growing burden of NCDs and the health inequalities affecting most disadvantaged populations.

Despite the strengths of this project, challenges are foreseen in its implementation. Maintaining an active partnership across numerous stakeholders with different competencies, roles, priorities, and expectations of the project outcomes is specifically built into the design, however this critical process requires time, dialogue and resources (29). Care will need to be taken to ensure equitable involvement of all partners with shared power and flexibility to make adjustments throughout the co-creation process. Also, in the current pandemic context, where stakeholders' organizations may already be overloaded, the project may place additional demands on some stakeholders and provide challenges for them to engage in full. The online format of most interactions within partnership during the indefinite COVID-19 pandemic will also require that partners adapt to new ways of connecting and working together, which may be particularly demanding in the phase of co-design of the interventions.

During the implementation of the co-designed solutions, it will be necessary to consider the degree of newness that the interventions will introduce to existing long-established practices in the organizations. Also, individuals with the greatest health literacy challenges are frequently the most socially vulnerable such that additional efforts by researchers are required to build trust and engage these groups in the interventions in a way that is meaningful and safe for them. Strategies, co-designed with and led by migrant communities, will need to be generated to ensure that local capacity building is undertaken in order to assure maximal participation of the key groups and beneficiaries. Another challenge is that, given the timeframe of the project, longer-term impacts and unexpected outcomes of the interventions may be difficult to assess.

## Conclusions

This project will give an equal voice to all stakeholders, including the end users, and will build on the existing local strengths to generate innovation. This protocol follows the Ophelia process, a principles-based health literacy-informed approach to co-designing services and systems that will lead to improved health, access and equity, and to enhanced quality of life and productive engagement in the society. It intends to generate a framework of best practice and suitable tools that can be adapted, considering differences and specificities (e.g., in terms of language, migration status, origins), to a range of contexts and populations with a migrant background, including in other European countries. The potential scalability of interventions to other similar contexts and populations may help produce evidence to further understand what common features, but also what specific contexts play a role in optimizing the efficacy of the interventions. Indeed, this will contribute to better understand how interventions can interact with different contexts to produce similar outcomes and what level of flexibility is needed to build, design and implement context-congruent interventions. Overall, the implementation of this protocol is expected to help advance the knowledge on how health literacy can improve health, well-being, and provide further understanding of its role as both a determinant and a response to reduce health inequalities among migrant communities and promote social integration.

## Ethics Statement

Ethical approval for the HLQ survey was obtained from the NOVA Medical School Ethics Committee. The participation in this study is voluntary. Informed consent is obtained from all participants. Anonymity and data confidentiality are guaranteed.

## Author Contributions

SD, ACF, and RHO contributed to the conceptualization of the project. SD, ACF, AGa, MJM, and ACM contributed to the design of the protocol. SD and AGa drafted the manuscript. All authors contributed to the Health Literacy Questionnaire study, reviewed the manuscript drafts, and approved the final draft of the manuscript.

## Conflict of Interest

The authors declare that the research was conducted in the absence of any commercial or financial relationships that could be construed as a potential conflict of interest.
