# Supplementary material for: Migrant Communities at the Center in Co-design of Health Literacy-Based Innovative Solutions for Non-communicable Diseases Prevention and Risk Reduction: Application of the OPtimising HEalth LIteracy and Access (Ophelia) Process
Source: Front Public Health. 2021 May 31;9:639405. doi: 10.3389/fpubh.2021.639405 (PMC8200814; doi:10.3389/fpubh.2021.639405)
Supplement: Supplementary file 1 [file Table_1.DOCX]

Table 1 – The Ophelia (OPtimising HEalth LIteracy and Access) principles (28).

| 1. Outcomes focused | Improved health and reduced health inequities |
| --- | --- |
| 2. Equity driven | All activities at all stages prioritize disadvantaged groups and those experiencing inequity in access and outcome |
| 3. Co-design approach | In all activities at all stages, relevant stakeholders engage collaboratively to design solutions |
| 4. Needs-diagnostic approach | Participatory assessment of local needs using local data |
| 5. Driven by local wisdom | Intervention development and implementation is grounded in local experience and expertise |
| 6. Sustainable | Optimal health literacy practice becomes normal practice and policy |
| 7. Responsiveness | Recognize that health literacy needs and the appropriate responses vary across individuals, contexts, countries, cultures and time |
| 8. Systematically applied | A multilevel approach in which resources, interventions, research and policy are organized to optimize health literacy |
